# Autophagy-Mediated Regulation of Lipid Metabolism and Its Impact on the Growth in Algae and Seed Plants

**DOI:** 10.3389/fpls.2019.00709

**Published:** 2019-06-04

**Authors:** Yushi Yoshitake, Hiroyuki Ohta, Mie Shimojima

**Affiliations:** ^1^ School of Life Science and Technology, Tokyo Institute of Technology, Yokohama, Japan; ^2^ Open Innovation Platform with Enterprises, Research Institute and Academia (OPERA), Japan Science and Technology Agency, Chiyoda, Japan

**Keywords:** autophagy, lipophagy, triacylglycerol, β-oxidation, carbon

## Abstract

Under nutrient starvation conditions, algae and seed-plant cells accumulate carbon metabolites such as storage lipids, triacylglycerols (TAGs), and starches. Recent research has suggested the involvement of autophagy in the regulation of carbon metabolites under nutrient starvation. When algae are grown under carbon starvation conditions, such as growth in darkness or in the presence of a photosynthesis inhibitor, lipid droplets are surrounded by phagophores. Indeed, the amount of TAGs in an autophagy-deficient mutant has been found to be greater than that in wild type under nitrogen starvation, and cerulenin, which is one of the inhibitors of fatty acid synthesis, induces autophagy. In land plants, TAGs accumulate predominantly in seeds and etiolated seedlings. These TAGs are degraded in peroxisomes *via* β-oxidation during germination as a source of carbon for growth without photosynthesis. A global analysis of the role of autophagy in *Arabidopsis* seedlings under carbon starvation revealed that a lack of autophagy enhances the accumulation of TAGs and fatty acids. In *Oryza sativa*, autophagy-mediated degradation of TAGs and diacylglycerols has been suggested to be important for pollen development. In this review, we introduce and summarize research findings demonstrating that autophagy affects lipid metabolism and discuss the role of autophagy in membrane and storage-lipid homeostasis, each of which affects the growth and development of seed plants and algae.

## Introduction

Most seed plants synthesize and store triacylglycerols (TAGs) in their seeds as a readily available source of carbon. TAGs in seeds are important for germination (Penfield et al., 2006). TAGs are also synthesized in the vegetative tissues of seed plants, but the amount is very low particularly under normal growth conditions (Chapman and Ohlrogge, 2012; Chapman et al., 2013). However, the amount of TAGs increases in the vegetative tissues under certain environmental stress conditions such as nutrient starvation, heat, or cold stresses (Mueller et al., 2015; Shimojima et al., 2015; Arisz et al., 2018). For the purpose of industrial oil production, many studies have attempted to clarify the regulatory mechanism of TAG metabolism in the seeds and vegetative tissues. However, the mechanism that regulates TAG synthesis and degradation is not fully understood.

Algae also accumulate TAGs in the cells under nutrient starvation (Hu et al., 2008; Kandilian et al., 2014; Slocombe et al., 2015; Taleb et al., 2016). A common feature of both plants and algae is that TAGs accumulate under environmental stress and are degraded, possibly owing to the need to supply carbon for cell growth. Moreover, TAGs are synthesized in the endoplasmic reticulum of both plants and algae, which is also the site for the synthesis of phospholipids and surface lipids. Here, we discuss current knowledge concerning two TAG degradation pathways in both plants and algae: β-oxidation and autophagy.

## Tag Degradation *Via* the β-Oxidation Pathway

**Figure 1:**
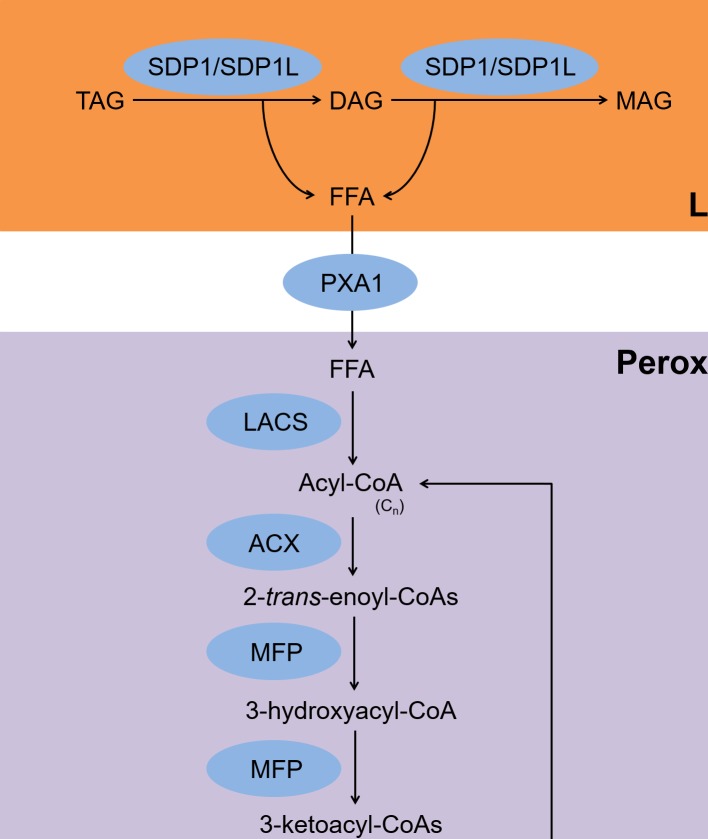
Scheme for TAG degradation *via* the β-oxidation pathway. FFAs are released from TAGs by SDP1/SDP1L and transferred to peroxisomes by PXA1. Next, LACS, ACX, MFP, and KAT produce acyl-CoA and acetyl-CoA. TAG, triacylglycerol; DAG, diacylglycerol; MAG, monoacylglycerol; FFA, free fatty acid; SDP1, sugar-dependent 1; SDP1L, SDP1-like; PXA1, peroxisomal ABC transporter 1; LACS, long-chain acyl-CoA synthase; ACX, acyl-CoA oxidase; MFP, multifunctional protein; KAT, 3-ketoacyl-CoA thiolase-2; LD, lipid droplet.

The fatty acid β-oxidation pathway is summarized in Figure 1. Free fatty acids (FFAs) that are released upon hydrolysis of TAGs are transported into peroxisomes where they are subsequently metabolized by the β-oxidation pathway in germinating seeds and vegetative tissues (Gerhardt, 1992). For a long time, β-oxidation was considered the only process of by which TAGs are degraded in plants (Gerhardt, 1992). During β-oxidation, the two TAG lipases, sugar-dependent 1 and sugar-dependent 1-like, associate with the surface of lipid droplets (LDs) and hydrolyze TAGs to produce FFAs and diacylglycerols (DAGs) (Eastmond, 2006; Kelly et al., 2011). Sugar-dependent 1 can also hydrolyze DAGs to produce FFAs and monoacylglycerols (Eastmond, 2006). The FFAs are transported to peroxisomes by the ATP-binding cassette transporter COMATOSE, also known as PXA1 (Zolman et al., 2001). In peroxisomes, acyl-CoAs are synthesized from the FFAs by the action of the peroxisomal long-chain acyl-CoA synthases 6 and 7 (Fulda et al., 2004). The acyl-CoAs are then converted to 2-*trans*-enoyl-CoAs by acyl-CoA oxidases 1–6 (Hooks et al., 1999; Rylott et al., 2003; Pinfield-Wells et al., 2005; Khan et al., 2012). The 2-*trans*-enoyl-CoAs are then hydrolyzed to produce 3-ketoacyl-CoAs *via* 3-hydroxyacyl-CoAs by the action of multifunctional protein 2 (Rylott et al., 2006). During the last step, the 3-ketoacyl-CoAs are hydrolyzed to produce acyl-CoAs and acetyl-CoAs by 3-ketoacyl-CoA thiolase-2, and the hydrolyzed acyl-CoAs are used as substrates for acyl-CoA oxidases (Germain et al., 2001). All of these reactions are required for seed germination when a carbon supply is lacking (Fulda et al., 2004; Pinfield-Wells et al., 2005; Eastmond, 2006; Footitt et al., 2007).

## Tag Degradation *Via* Autophagy in Algae and Seed Plants

Autophagy is one of the major degradative systems used for quality control of proteins and organelles (Klionsky and Ohsumi, 1999; Lilienbaum, 2013). There are 15 “core” ATG genes, namely *ATG1–10*, *ATG12–14*, *ATG16,* and *ATG18*, that are required for the formation of the autophagosomal membrane. In particular, covalent modification of the ATG proteins with ubiquitin is a critical event in autophagosome formation (Mizushima et al., 2011). ATG4, one of the cysteine proteases, cleaves ATG8 at a specific sequence in its C-terminal region (Kirisako et al., 2000). ATG7 is an E1-like enzyme that activates ATG8 and ATG12 (Mizushima et al., 1998; Ichimura et al., 2000). Then, ATG3 mediates the conjugation of phosphatidylethanolamine to ATG8, and ATG10 mediates the covalent conjugation of ATG12 with ATG5 (Mizushima et al., 1998; Yamaguchi et al., 2010). ATG12-ATG5 then interacts with ATG16 and ATG12-ATG5-ATG16 complexes form a dimer (Mizushima et al., 1998; Kuma et al., 2002). Recent studies have revealed that autophagy is not only a non-selective bulk degradation pathway but also is involved in the selective elimination or degradation of components such as aberrant aggregated proteins, dysfunctional organelles, invading pathogens, and LDs (Yuan et al., 1997; Roberts et al., 2003; Lemasters, 2005; Bernales et al., 2006; Izumi et al., 2010, 2017; Yoshimoto et al., 2010; Schuck et al., 2014; Elander et al., 2018). TAGs are produced in the endoplasmic reticulum and then become enclosed by LDs after being exported to the cytoplasm, and each LD is surrounded by a monolayer of phospholipids and surface proteins. It has been proposed that selective autophagy takes part in the degradation of LDs (Singh et al., 2009) *via* a mechanism called “lipophagy,” which is observed in seed plants and algae (Kurusu et al., 2014; Zhao et al., 2014; Avin-Wittenberg et al., 2015; Schwarz et al., 2017; Elander et al., 2018).

In the unicellular model alga *Micrasterias denticulata*, autophagy was observed under salt stress and cadmium stress (Affenzeller et al., 2009; Andosch et al., 2012; Lütz-Meindl, 2016). Upon exposure of *Micrasterias* cells to salt stress, electron microscopy revealed that the dictyosomes and some unidentifiable organelles were surrounded by a double membrane (Affenzeller et al., 2009), and upon exposure to cadmium stress, autophagosomes, including Golgi remnants, vesicles, and cytoplasmic portions, were observed (Andosch et al., 2012). Transmission electron microscopy studies suggested that autophagy might be induced in response to carbon starvation in Micrasterias (Schwarz et al., 2017). In *Micrasterias* cells, LDs are formed and accumulated in chloroplasts, and carbon starvation can lead to the displacement of LDs from chloroplasts to the cytoplasm in the isthmus region of cells (Schwarz et al., 2017). The LDs are engulfed by endoplasmic reticulum-derived double membranes, which resembles autophagy (Schwarz et al., 2017). In another alga, *Auxenochlorella protothecoides*, autophagy is induced during the heterotroph-to-autotroph transition, and LDs in heterotrophic cells are degraded in the vacuole (Zhao et al., 2014). This process is similar to microautophagy, but not macroautophagy, because LDs seem to be engulfed directly by the vacuole. These studies suggested that, under carbon starvation, LDs and their constituent TAGs are degraded by lipophagy or autophagy-like processes. Moreover, in mutants of *Chlamydomonas reinhardtii* that are deficient for autophagy (*atg8*), TAG degradation was slower than that measured in wild-type cells under nitrogen-replete conditions (Kajikawa et al., 2019). It was also shown that vacuolar lytic function is needed for the synthesis of TAGs and the formation of LDs in nitrogen- or phosphate-starved Chlamydomonas cells using concanamycin A, a vacuolar ATPase inhibitor, which blocks autophagic flux (Couso et al., 2018). In general, autophagy mutants accumulate more TAGs than do wild-type cells under sulfate starvation (Kajikawa et al., 2019). Under nitrogen starvation, cerulenin, which is one of the fatty acid synthase inhibitors, activates autophagy and reduces TAG content in wild-type cells (Heredia-Martínez et al., 2018). These findings suggest that not only carbon starvation but also other stresses can induce TAG degradation *via* lipophagy in algae.

In seed plants, TAG degradation is required for seed germination (Penfield et al., 2006). Although β-oxidation is essential for TAG degradation during germination, autophagy is the primary mechanism for TAG degradation during seedling growth after germination. When seeds were sown in 1/2× Murashige and Skoog medium without sucrose and then grown in darkness for 7 days, autophagy mutants of *Arabidopsis thaliana* (*atg5*, *atg7*) are able to germinate but had shorter hypocotyls than wild type (Avin-Wittenberg et al., 2015). However, the hypocotyl lengths of autophagy mutants grown on the medium containing sucrose were similar to that of wild type (Avin-Wittenberg et al., 2015), clearly indicating that autophagy is essential for providing sufficient carbon for the initial growth of seedlings under carbon starvation. Several β-oxidation mutants such as *sdp1*, *acx1-1 acx2-1*, *lacs6 lacs7*, and *kat2-1* also have shorter hypocotyls than wild type grown under carbon starvation, suggesting that β-oxidation is also involved in TAG degradation during the initial growth of seedlings (Fulda et al., 2004; Pinfield-Wells et al., 2005; Eastmond, 2006; Footitt et al., 2007). These findings clearly show that both autophagy and β-oxidation are required for TAG degradation until seedlings become established and are able to carry out photosynthesis. However, it remains unclear how autophagy and β-oxidation each contribute to seedling maturation.

In *Arabidopsis*, lipophagy has been observed during initial growth but not in vegetative tissues (Avin-Wittenberg et al., 2015). In *O. sativa*, however, lipophagy occurs in tapetal cells under normal growth conditions, and fewer TAGs and DAGs are present in autophagy mutants (*atg7*) than in wild type (Kurusu et al., 2014). Moreover, pollen maturation is inhibited in autophagy mutants of *O. sativa* (Kurusu et al., 2014). These findings indicate that lipophagy can be induced under normal growth conditions and is required for reproductive development in rice.

## Conclusion

In algae, β-oxidation has an important role in carbon management during the day and night periods (Kong et al., 2017). In *Auxenochlorella*, however, lipophagy is induced during the heterotroph-to-autotroph transition (Zhao et al., 2014). These data also suggest that lipophagy and β-oxidation are differentially regulated during TAG degradation in algae. Moreover, in *Chlamydomonas reinhardtii*, the activation of autophagy has a negative effect on the accumulation of TAGs and the formation of LDs (Heredia-Martínez et al., 2018; Kajikawa et al., 2019). In seed plants, TAGs and FFAs are degraded by β-oxidation in peroxisomes during the germination of plant seeds (Gerhardt, 1992; Penfield et al., 2006). However, in *Arabidopsis*, lipophagy is involved in early seedling development but is not essential for seed germination. In the case of *Oryza*, however, pollen maturation is altered in autophagy mutants, suggesting that lipophagy is required for normal reproductive development in some seed plants (Kurusu et al., 2014). However, the specific proteins that control the induction of lipophagy remain unknown in both algae and land plants. In case of liver cells, the small GTPase Rab7 is activated upon nutrient starvation to recruit autophagosomes (Schroeder et al., 2015). Thereafter, the complex composed of Rab10-EH domain-binding protein 1 and ATPase EH domain containing 2 promotes the expansion of the autophagic membrane, and the membrane then can engulf LDs (Li et al., 2016). Moreover, Dynamin 2 is required for vesiculation of autolysosomal tubules, which contain LDs (Schulze et al., 2013). On the other hand, ethanol-induced lipophagy requires SQSTM1, which is a major autophagy adaptor (Wang et al., 2017). When liver cells are stimulated with ethanol, perilipin 1, which is a major protein on LDs, colocalizes with SQSTM1 and promotes ethanol-induced lipophagy (Wang et al., 2017). To attain a better understanding of the physiological functions of lipophagy in seed plants and algae, clarification of the regulatory mechanism of lipophagy induction and its coordination with β-oxidation will require the identification of the lipophagy players and an analysis of knockout mutants.

## Author Contributions

All authors listed have made a substantial, direct, and intellectual contribution to the work and approved it for publication.

### Conflict of Interest Statement

The authors declare that the research was conducted in the absence of any commercial or financial relationships that could be construed as a potential conflict of interest.
